# Design and Experimental Test of Rope-Driven Force Sensing Flexible Gripper

**DOI:** 10.3390/s24196407

**Published:** 2024-10-03

**Authors:** Zuhao Zhu, Yufei Liu, Jinyong Ju, En Lu

**Affiliations:** 1 School of Artificial Intelligence, Anhui Polytechnic University, Wuhu 241000, China; 13485798953@139.com (Z.Z.); junjy@ahpu.edu.cn (J.J.); 2 School of Mechanical and Automotive Engineering, Anhui Polytechnic University, Wuhu 241000, China; 3 School of Agricultural Engineering, Jiangsu University, Zhenjiang 212013, China; jsluen@163.com

**Keywords:** flexible gripper, force sensing, rope-driven, control system, experimental test, robotic grasping system

## Abstract

Robotic grasping is a common operation scenario in industry and agriculture, in which the force sensing function is a significant factor to achieve reliable grasping. Existing force sensing methods of flexible grippers require intelligent materials or force sensors embedded in the flexible gripper, which causes such problems of higher manufacturing requirements and contact surface properties changing. In this paper, a novel rope-driven force sensing flexible gripper is designed based on the fin-shaped gripper structure, which can realize the grasping sensing functions of contact nodes and contact forces without the need for force sensors. Firstly, the rope-driven force sensing flexible gripper is designed, including the driving unit, the transmission part, the gripper unit, and the force sensing unit. The force sensing unit and the gripper unit are connected by rope, and the prototype of the rope-driven force sensing flexible gripper is completed. Secondly, a force sensing algorithm and control system based on finite element method and grasping geometric relationship are designed to realize the rope-driven force sensing flexible gripper grasping control and sensor data acquisition and processing. Finally, the experimental system of the rope-driven force sensing flexible gripper is built, and the grasping experimental tests of objects with different diameters and different contact nodes are carried out to verify the force sensing function of the rope-driven force sensing flexible gripper. The force sensing flexible gripper designed in this paper can provide a new idea for the design and force sensing method of intelligent robotic grasping system in robotic teaching, scientific research, and industrial applications.

## 1. Introduction

Robot grasping has the advantages of high efficiency and lower labor cost, which has been widely used in industrial and agricultural fields [1,2,3]. The end-effector for the grasping operation needs the functions of visual sensing [4,5,6] and force sensing [7], especially for the soft and fragile objects which needs a flexible gripper [8,9]. In recent years, many scholars have conducted related research on the flexible gripper [10,11,12]. Liu [13] combined origami technology with nonlinear topology optimization method to design a flexible gripper based on origami teeth, which could grip objects of different shapes. Wang [14] designed a flexible gripper using thick plate origami, which could complete the grasping when it was disturbed by external forces, but the grasping load was limited due to the influence of its own materials. Li [15] proposed a kind of untethered pneumatic gripper with high load based on the principle of biotin spiral winding, which has a biomimetic soft winding structure driven by PAM to realize the grasping of high-quality heavy objects. Liu [16] designed a constant force flexible gripper, which used a flexible slider crank mechanism to connect with the gripper and generated a constant gripping force during grasping, which was robust to environmental interference. Hao [17] designed a soft actuator with a fingerprint texture based on the anatomical structure of human finger, which increased the contact area with the object and improved the grasp ability. Wang [18] designed a soft pneumatic gripper based on animal claws and human fingers, which overcame the shortcomings of single bending direction and limited obstacle avoidance ability of grasping, improving the reliability of grasping. Fan [19] fabricated flexible double-layer ETAs based on graphite paper and PI film, and the flexible brake could achieve effective bending and driving in a low-pressure environment. Ji [20] and Nie [21] designed a three-finger flexible actuator based on water hydraulic drive, control of high-pressure water entry and exit to achieve flexible opening and closing grasping in the ocean.

A flexible gripper has obvious advantages in grasping flexible objects, and the force sensing function is required for the complete flexible grasping of soft objects. Jin [22] designed a two-finger gripper based on fin-shaped flexible gripper. The pressure sensor and bending sensor were placed on the inner and outer surface of each flexible gripper, and the force-sensitive resistance sensor was pasted on the inner surface of the flexible gripper to detect pressure and deformation thus realizing the evaluation of kiwi fruit hardness. Chen [23] designed a flexible pneumatic gripper integrated with three fingers. The pneumatic pressure sensor was used to detect the grasping force, and the bending sensor was placed on the surface of the gripper to detect the grasping position. Ren [24] and Dilibal [25] adopted 3D printing to make a flexible gripper and placed the embedded soft force sensor on the contact surface of the gripper to realize the force perception function while grasping. However, when the sensor was placed on the surface of the gripper [26,27,28], the grasping performance of the flexible gripper was affected. Lan [29] and Wang [30] used smart material shape memory alloy (SMA) to fabricate a flexible gripper to realize force sensing, but there were limitations in grasping the load. Xie [31], Wang [32], Wang [33] made a flexible gripper with tactile or visual perception through sensors, but they could only be used on specific grippers, which has limitations in the use scenario. Aiming at the above problems, Zhang [34] and Xu [35] optimized the design of the universal flexible gripper and added a force sensing module to calculate the grasping force of the flexible gripper through bending sensors or vision systems to realize the force sensing function. Yao [36] and Shan [37] modeled the universal flexible gripper based on the finite element method and the principle of virtual work to reveal the calculation method of contact force sensing of flexible gripper. However, the above force sensing methods need to input the contact points and other conditions before calculation, and the calculation process is more complicated.

It can be seen that the existing force sensing implementation of a flexible gripper requires additional embedded smart materials or force sensors, which causes problems such as high manufacturing accuracy requirements, complex sensor installation, and changing contact surface properties. With a focus on these deficiencies, this paper proposes a rope-driven force sensing flexible gripper and its control system, which can realize the sensing function of grasping contact nodes and contact forces without the use of force sensors thus avoiding the problems existing in the traditional flexible gripper using force sensors for force sensing. The contents of this paper are as follows: In Section 2, the structure design and prototype development of the rope-driven force sensing flexible gripper are presented. In Section 3, the design of the force sensing strategy of rope-driven force sensing flexible gripper is discussed. In Section 4, a rope-driven force sensing flexible gripper experimental platform is built to verify the force sensing function of the flexible gripper through experimental tests of grasps with different diameters and different contact nodes. Finally, the conclusions drawn from the study are summarized in Section 5.

## 2. Structure Design and Prototype Development

**Figure 1:**
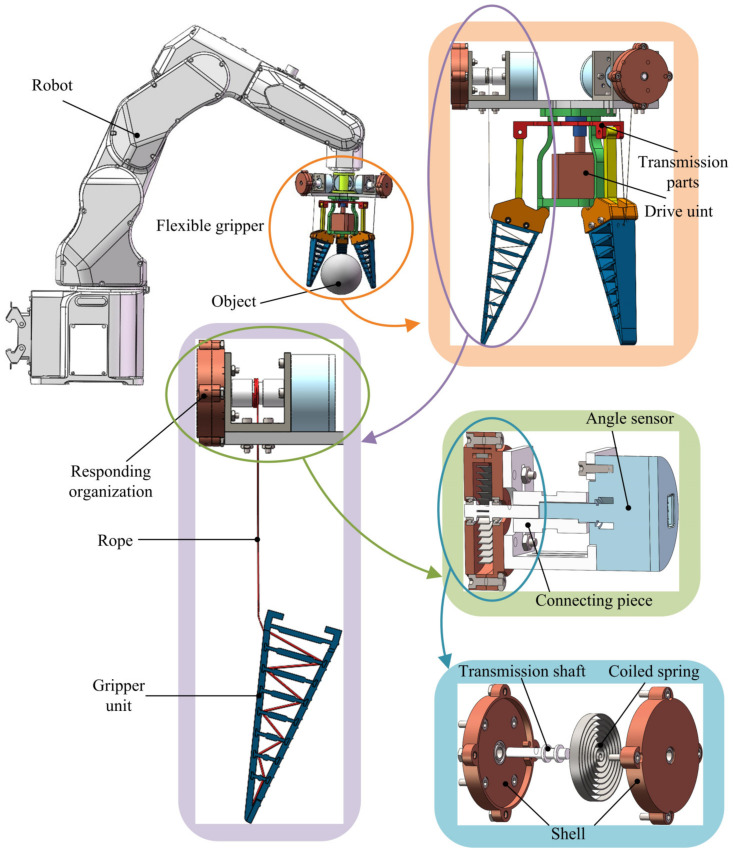
Overall structure of rope-driven force sensing flexible gripper.

**Figure 2:**
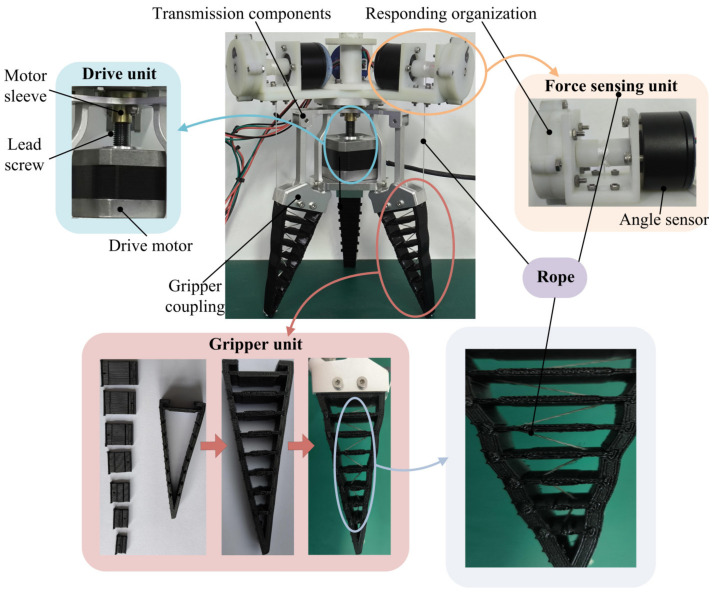
Developed prototype of rope-driven force sensing flexible gripper.

In this paper, a rope-driven force sensing flexible gripper is designed, as shown in Figure 1 and Figure 2, which is mainly composed of a driving unit, a transmission parts, a gripper unit, and a force sensing unit. Among them, the drive unit has a lead screw; the lead screw is installed with a threaded motor sleeve; the motor sleeve and the transmission parts are a fixed connection; the force sensing unit is mainly composed of an angle sensor, a connecting piece, and a responding organization; and the responding organization includes a transmission shaft, a shell, and a coiled spring. The force sensing unit is installed and fixed at the end of the flexible gripper, and the force sensing unit and the gripper unit are connected by rope.

In the process of use, the flexible gripper is installed at the end of the robot through the flange. When grasping objects, the lead screw on the driving unit rotates to drive the motor sleeve and the transmission parts fixed with the motor sleeve to move up and down. The transmission parts drive the gripper unit to move through the gripper coupling, then the terminal gripper unit moves in the opposite direction of the force. The rotation of the angle sensor of the rope-driven force sensing unit and combined force sensing algorithm realize the detection of the contact force of the flexible gripper.

According to the designed structure of a rope-driven force sensing flexible gripper, a prototype of the rope-driven force sensing flexible gripper is developed. The flexible gripper drive unit adopts a two-phase hybrid stepper motor, and the transmission structure is composed of connecting rods; 3D printing technology is used to fabricate and process the rope-driven force sensing flexible gripper force sensing unit. A TPU material is used for the printing of the gripper unit. The gripper unit is composed of seven removable crossbeams and the gripper shell. There are holes at the edge of each crossbeam, and the holes of the adjacent crossbeams are opposite of each other. One end of the rope is fixed at the bottom of the gripper shell, and the other end is passed through the holes of the crossbeam in turn and then wound on the force sensing unit through the holes of the gripper shell. The connecting rope has the characteristics of inelasticity and good firmness. The GRM angle sensor is used in the force sensing unit. For the resetting of the GRM angle sensor after the measurement is completed, the device equipped with a coiled spring is used as the responding organization. Figure 2 shows the developed rope-driven force sensing flexible gripper prototype.

## 3. Force Sensing Strategy

### 3.1. Relationship Matrix

**Figure 3:**
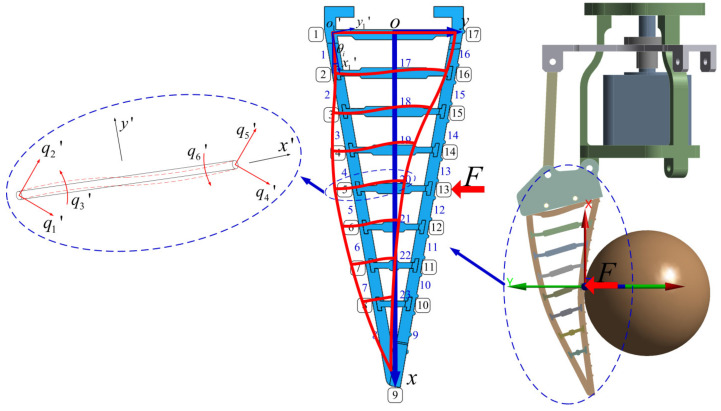
Force analysis of rope-driven force sensing flexible gripper grasping.

The force analysis during the grasping process of the flexible gripper is shown in Figure 3. Based on the finite element method, the gripper unit is discretized and divided into elements. The following assumptions are made: The gripper unit conforms to the linear elastic characteristics within the effective grasp range; the divided elements are regarded as the synthesis of rod elements and beam elements; and the joints of elements do not contain elastic hinges and rotating joints. The divided elements and nodes are numbered such that the black numbers with boxes are node numbers and the blue numbers are elements numbers. Since each end of the rod element has one degree of freedom of movement, each end of the beam element has one degree of freedom of movement and one degree of freedom of rotation. So, each element in the gripper unit has three degrees of freedom at the end, respectively.

According to the finite element method, the force–deformation equation can be described as follows:

(1) $$Q=K^{-1}F,$$

where *K*, *Q* and *F* are the global stiffness matrix (*K*) of the gripper unit, the deformation vector of each node (*Q*), and the force vector of each node (*F*), respectively.

Then, global stiffness matrix *K* can be further expressed as follows:

(2) $$K=\sum L_{i}^{T}K_{i}L_{i},$$

The element stiffness matrix obtained by reassembling the stiffness matrix of the rod element and beam element is defined as *K_i_*, which can be expressed as follows:

(3) $$K_{i}=\left[\begin{array}{cccccc} \frac{EA}{l_{e}} & 0 & 0 & \frac{-EA}{l_{e}} & 0 & 0\\ 0 & \frac{12EI}{l_{e}^{3}} & \frac{6EI}{l_{e}^{2}} & 0 & \frac{-12EI}{l_{e}^{3}} & \frac{6EI}{l_{e}^{2}}\\ 0 & \frac{6EI}{l_{e}^{2}} & \frac{4EI}{l_{e}} & 0 & \frac{-6EI}{l_{e}^{2}} & \frac{2EI}{l_{e}}\\ \frac{-EA}{l_{e}} & 0 & 0 & \frac{EA}{l_{e}} & 0 & 0\\ 0 & \frac{-12EI}{l_{e}^{3}} & \frac{-6EI}{l_{e}^{2}} & 0 & \frac{12EI}{l_{e}^{3}} & \frac{-6EI}{l_{e}^{2}}\\ 0 & \frac{6EI}{l_{e}^{2}} & \frac{2EI}{l_{e}} & 0 & \frac{-6EI}{l_{e}^{2}} & \frac{4EI}{l_{e}} \end{array}\right],$$

where *E*, *A*, *l_e_* and *I* are the Young’s modulus, cross-sectional area, length, and moment of inertia of the element, respectively.

The transformation matrix is defined as *L_i_*, which can be shown as follows:

(4) $$L_{i}=\left[\begin{array}{cccccc} c\theta_{i} & s\theta_{i} & 0 & 0 & 0 & 0\\ -s\theta_{i} & c\theta_{i} & 0 & 0 & 0 & 0\\ 0 & 0 & 1 & 0 & 0 & 0\\ 0 & 0 & 0 & c\theta_{i} & s\theta_{i} & 0\\ 0 & 0 & 0 & -s\theta_{i} & c\theta_{i} & 0\\ 0 & 0 & 0 & 0 & 0 & 1 \end{array}\right],$$

where *θ_i_* is the inclined angle between the *x_i’_*-axis of the local coordinate system and the *x*-axis of the global coordinate system. *cθ_i_ = cosθ_i_; sθ_i_ = sinθ_i_*

According to the actual installation of the gripper unit, node 1 and node 17 are taken as fixed constraints. By substituting the force vector (*F*) and the global stiffness matrix (*K*) in Equation (1), the displacement vector (*Q*) containing the displacements and rotation angles of all nodes can be obtained. The displacement vector *Q* can be represented as follows:

(5) $$Q=(d_{1}^{x},d_{1}^{y},\alpha_{1},d_{2}^{x},d_{2}^{y},\alpha_{2},d_{3}^{x},d_{3}^{y},\alpha_{3},\cdots \cdots d_{i}^{x},d_{i}^{y},\alpha_{i}\cdots \cdots ,d_{17}^{x},d_{17}^{y},\alpha_{17}),$$

In summary, through the force–deformation equation obtained by the finite element method, the corresponding displacement and angle of all nodes of the gripper unit can be obtained when a certain node is stressed.

When the *i*th node is under force, the force–displacement equation between the displacement of the terminal point and the contact force can be further obtained according to Equation (1) as follows:

(6) $$F_{i}=k_{i}^{9}s_{i}^{9},$$

where $k_{i}^{9}$ is the correspondence between the force on the ith node (*F_i_*) and the displacement ($s_{i}^{9}$) of the terminal point under the global coordinate system (*o-xy*) of the gripper unit. The value of $k_{i}^{9}$ is determined by the position of the force node *i* and the global stiffness matrix *K*.

### 3.2. Determination of Contact Force

**Figure 4:**
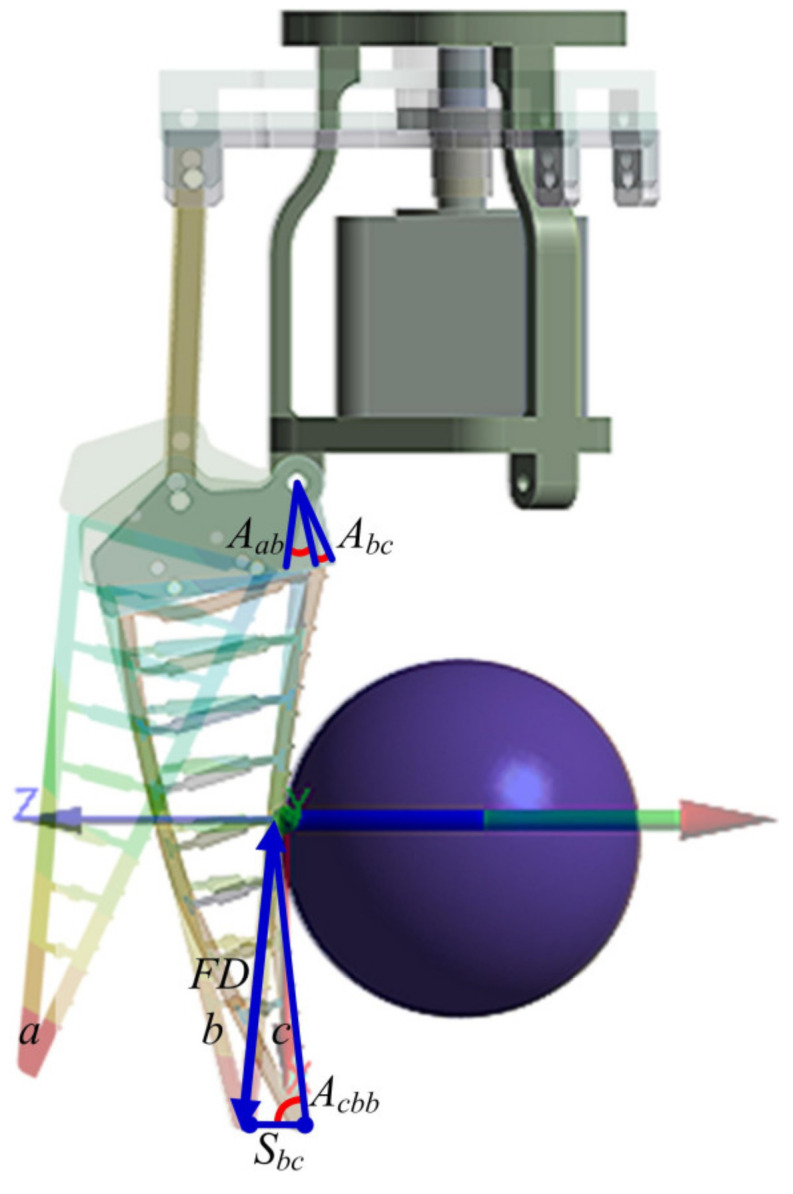
Rope-driven force sensing flexible gripper grasping process.

As shown in Figure 4, the grasping process of the rope-driven force sensing flexible gripper can be divided into the following three states: the initial moment (*a*), the first contact moment (*b*) and the completion moment (*c*). The process of force sensing is as follows.

First, determine the contact node. According to the geometric relationship of different grasping states, the following definitions are made: The distance between the contact node and the terminal point is *FD*. The displacement of the terminal point and the rotation angle of the top from the first contact moment to the completion moment is *S_bc_* and *A_bc_*. The angle between the line between the terminal point at the completion moment and the first contact moment and the line between the terminal point at the completion moment and the contact is *A_cbb_*. *FD* can be expressed as follows:

(7) $$FD=\frac{\sin(A_{cbb})S_{bc}}{\sin(A_{bc})},$$

According to the structural parameters and node division of the gripper unit shown in Figure 3, the contact node (*i*) during the grasping of the rope-driven force sensing flexible gripper can be obtained.

Secondly, the displacement of the terminal point ($s_{i}^{9}$) in the *o-xy* coordinate system is judged. According to the geometric relationship between the first contact moment and the completion moment, the terminal point displacement (*S_bc_*) measured by the sensor in the global coordinate system (*O-XY*) of the rope-driven force sensing flexible gripper is transformed into the terminal point displacement ($s_{i}^{9}$) in the *o-xy* coordinate system. The following definition is formed: The rotation angle of the top from the initial moment to the first contact moment is *A_ab_*. According to the structural parameters of the rope-driven force sensing flexible gripper, the length of the gripper unit is obtained as *l*. $s_{i}^{9}$ can be expressed as follows:

(8) $$s_{i}^{9}=\frac{l(s\theta_{2}-s\theta_{1})}{s\theta_{3}}-s_{m}+s\theta_{3}S_{bc},$$

where *s_m_* = *l*[*sθ*_3_ (*cθ*_2_ − *sθ*_2_) + *sθ*_2_ − *sθ*_1_], *θ*_1_ = 64.14° − *A_ab_*, *θ*_2_ = 64.14° + *A_ab_* + *A_b__c_*, θ_3_ = 54.14° + *A_ab_* + *A_b__c_*.

Finally, by substituting Equations (7) and (8) into Equation (6), the force Fi of the contact node of the rope-driven force sensing flexible gripper can be obtained.

**Figure 5:**
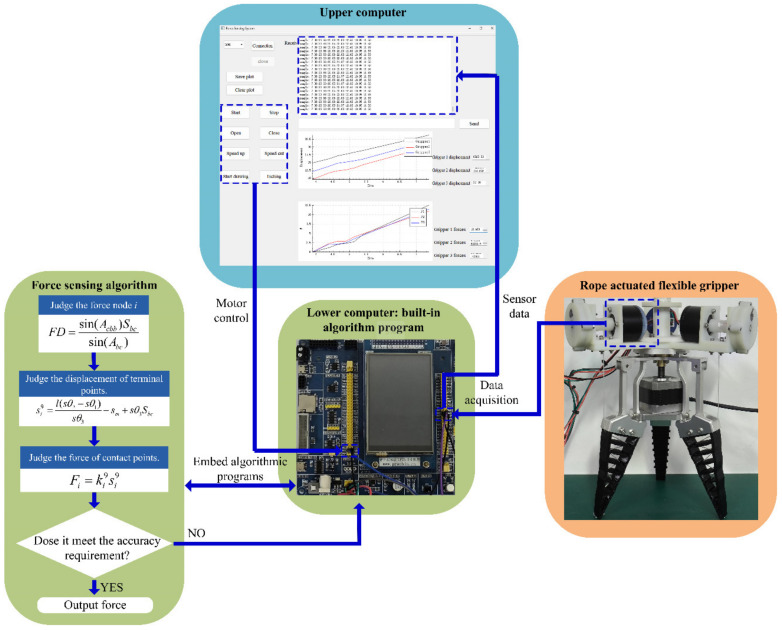
The force sensing process of the rope-driven force sensing flexible gripper.

Figure 5 shows the force sensing process of the rope-driven force sensing flexible gripper. In the actual process, the start signal is sent to the lower computer by clicking the button of the upper computer. The lower computer then analyzes the signal and controls the rope-driven force sensing flexible gripper closure. When the rope-driven force sensing flexible gripper grasps the object, the lower computer receives the data transmitted by the angle sensor and determines the force of the contact node of the rope-driven force sensing flexible gripper with its built-in force sensing program. Data processing is carried out inside the lower computer; the processed data are uploaded, plotted, and displayed in the upper computer.

## 4. Experimental Study

### 4.1. Experimental System

**Figure 6:**
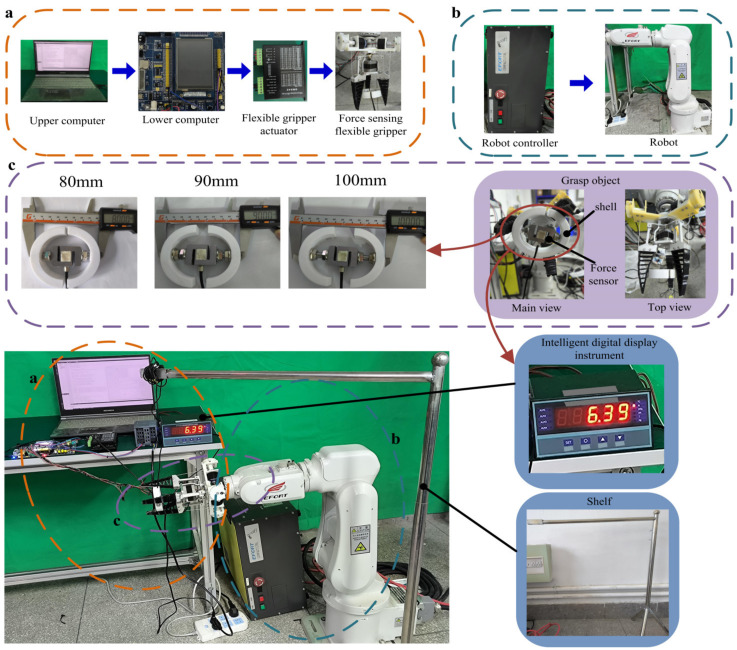
Experimental system of the rope-driven force sensing flexible gripper.

In order to verify the grasping contact force sensing function of the rope-driven force sensing flexible gripper, the experimental system of the rope-driven force sensing flexible gripper is set up as shown in Figure 6. The experimental system includes a robot, the rope-driven force sensing flexible gripper, and an object to be grasped. The robot uses a 6-DOF industrial robot, while the gripper unit is composed of seven removable crossbeams of the rope-driven force sensing flexible gripper, which is made of a flexible TPU material with good linear elasticity through 3D printing. The stepping motor is used as the driving unit to realize the opening and closing action of the rope-driven force sensing flexible gripper. The force sensing unit is made of a 3D printed resin material, and includes a angle sensor (GRM, produced by Wheel Technology (Dongguan) Co., LTD, Dongguan, China)and responsive architecture with a coiled spring to realize the reset of the angle sensor, while the flexible gripper is connected with the force sensing unit through a rope. In order to compare and verify the grasping contact force of the rope-driven force sensing flexible gripper, a grasping object simulation device with integrated force sensor is designed, and the actual grasping contact force can be obtained at the same time during the grasping process. In this paper, force sensor (HZC-MS3, produced by Bengbu Chengying sensor Co., LTD, Bengbu, China) and digital display instrument (MCK-Z-I, produced by Bengbu Chengying sensor Co., LTD, Bengbu, China) were used. The sensor was placed in a shell made by 3D printing. During grasping, three gripper units grasped the shell, and the sensor in the shell was aligned with the gripper unit to measure the grasping force. The MCU with STM32F103 chip (produced by Shenzhen Puzhong Technology Co., LTD, Shenzhen, China) as the core was used as the lower computer to build the control system, and QT was used to build the upper computer for control and data display.

### 4.2. Results Analysis

According to the experimental system, the experimental verification of the rope-driven force sensing flexible gripper grasping force sensing strategy was carried out. Three different diameter objects were selected for the grasping experiments, and three grasping contact nodes were selected for each diameter object. In the grasping process, the force sensing unit transmitted the data to the lower computer for node judgment and contact point force calculation, then the data results were transmitted to the upper computer for the data and drawing display. The experimental results are shown below.

**Figure 7:**
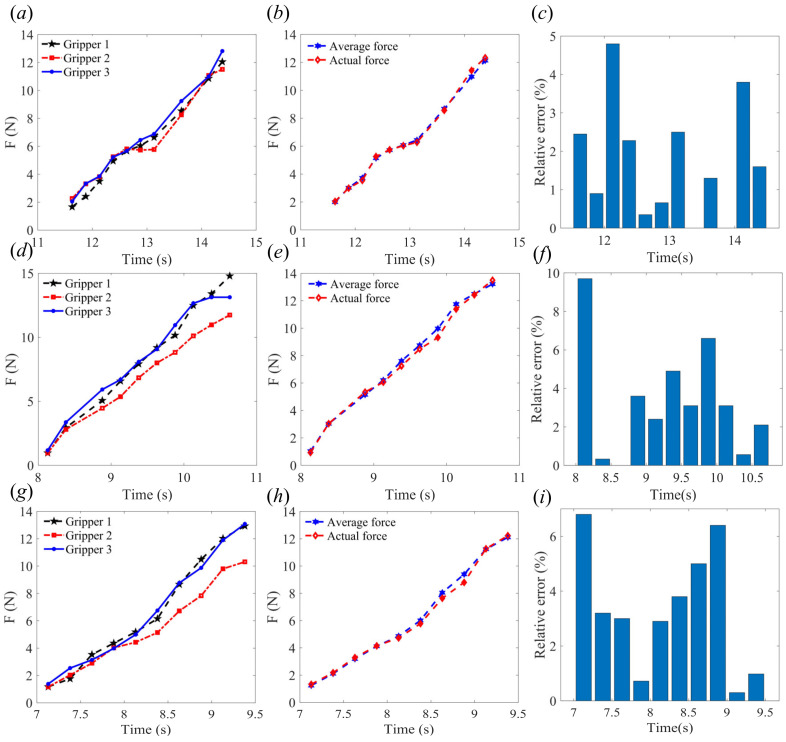
Experimental test results (diameter 80 mm): (**a**) grasping force of node 11; (**b**) average force compared with actual force of node 11; (**c**) relative error of grasping force of node 11; (**d**) grasping force of node 13; (**e**) average force compared with actual force of node 13; (**f**) relative error of grasping force of node 13; (**g**) grasping force of node 14; (**h**) average force compared with actual force of node 14; and (**i**) relative error of grasping force of node 14.

As shown in Figure 7, the rope-driven force sensing flexible gripper to grasp objects of 80mm diameter at node 11, node 13, and node 14, respectively. Figure 7a shows the force sensing results of the three gripper units when grasping the object at contact node 11, while Figure 7b shows the comparison between the average force of the three gripper units and the actual force, and Figure 7c shows the relative error between the average force of the three gripper units and the actual force. Figure 7d–f show the results of the three gripper units grasping the object at node 13, Figure 7g–i show the results of the three gripper units grasping the object at node 14. It can be seen that in the initial stage of the grasp, the relative error is large while the absolute error is small, while in the end stage of the grasp, the absolute error is large but the relative error is small. The effect of force sensing in the middle stage is better than in the initial stage and the end stage when the rope-driven force sensing flexible gripper performs force sensing.

**Figure 8:**
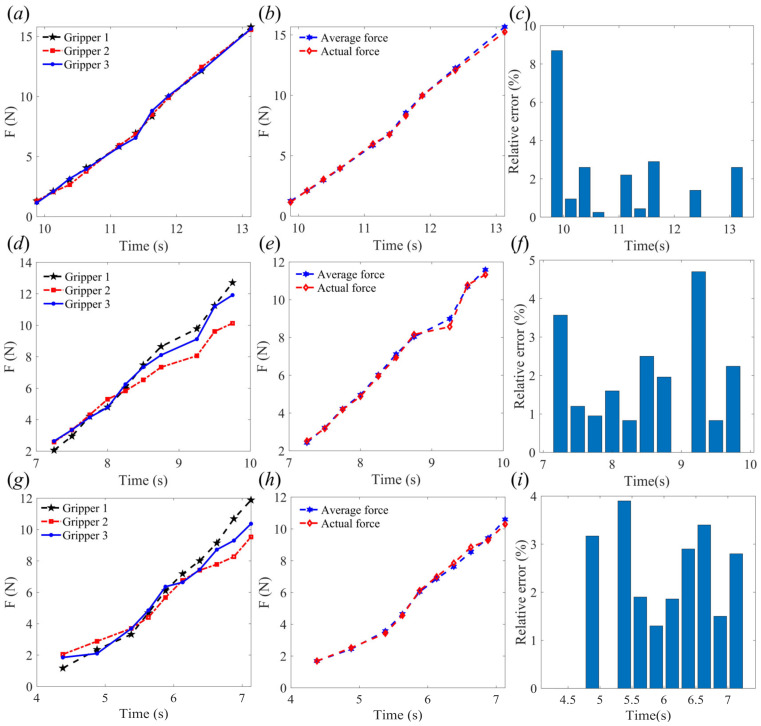
Experimental test results (diameter 90 mm): (**a**) grasping force of node 11; (**b**) average force compared with actual force of node 11; (**c**) relative error of grasping force of node 11; (**d**) grasping force of node 13; (**e**) average force compared with actual force of node 13; (**f**) relative error of grasping force of node 13; (**g**) grasping force of node 14; (**h**) average force compared with actual force of node 14; (**i**) relative error of grasping force of node 14.

As shown in Figure 8, the rope-driven force sensing flexible gripper to grasp objects of 90mm diameter at node 11, node 13, and node 14, respectively. Figure 8a,d,g show the force sensing results of the three gripper units when grasping the object at node 11, node 13, and node 14, respectively. Figure 8b,e,h show the comparison between the average force and the actual force when the three gripper units grasp the object at node 11, node 13, and node 14, respectively. Figure 8c,f,i show the relative errors between the average force and the actual force when the three gripper units grasp the object at node 11, node 13, and node 14, respectively. It can be seen that the rope-driven force sensing flexible gripper has a small error in the initial stage of grasping at node 14, but there is a large error between the force sensing of the three gripper units when grasping at nodes 13 and 14. When grasping at node 11, the force sensing effect is the best, and it can be seen that the force sensing effect in the middle stage is better than in the initial stage and the end stage.

**Figure 9:**
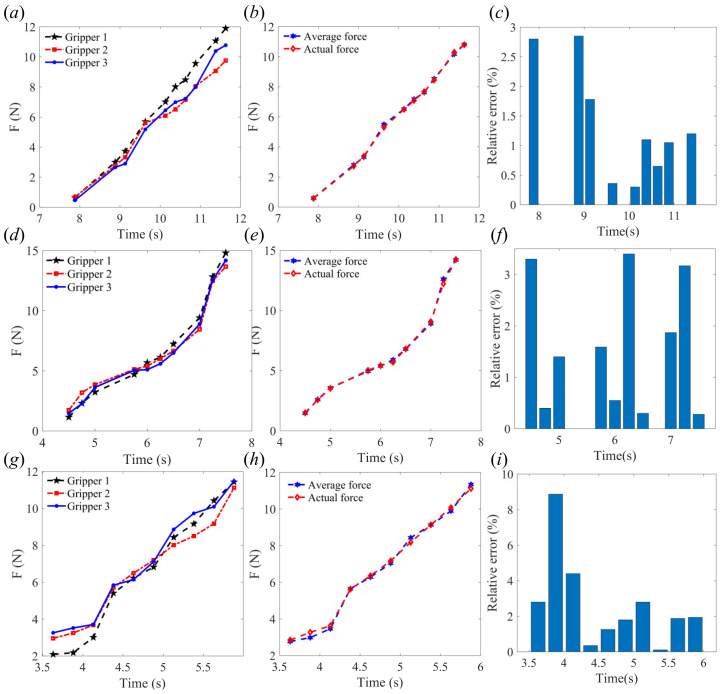
Experimental test results (diameter 100 mm): (**a**) grasping force of node 11; (**b**) average force compared with actual force of node 11; (**c**) relative error of grasping force of node 11; (**d**) grasping force of node 13; (**e**) average force compared with actual force of node 13; (**f**) relative error of grasping force of node 13; (**g**) grasping force of node 14; (**h**) average force compared with actual force of node 14; (**i**) relative error of grasping force of node 14.

As shown in Figure 9, the rope-driven force sensing flexible gripper to grasp objects of 100 mm diameter at node 11, node 13, and node 14, respectively. Figure 9a–c show the force sensing results, the comparison between the average force and the actual force and the relative error between the average force and the actual force of the three gripper units when grasping the object at node 11, respectively. The force sensing results, the comparison between the average force and the actual force as well as the relative error between the average force and the actual force of the three gripper units when grasping the object at node 13 are shown in Figure 9d–f, respectively. Figure 9g–i demonstrate the grasping results of the three gripper units when grasping the object at node 14. It can be seen that the absolute error of force sensing of the rope-driven force sensing flexible gripper is larger at the end stage, and the relative error is larger at the initial stage. The absolute error in the middle stage is smaller, and the force sensing effect in the middle stage is better than in the initial stage and the end stage.

The flexible gripper grasped objects with different diameters at different nodes. The data are collected and processed in the lower computer and displayed in the upper computer. The average error of force sensing by the force sensing strategy is about 2.3%. It can be seen that the absolute error between the three gripper units is small when grasping at node 11, and the force sensing effect is best in the middle stage of grasping. The results show that the force sensing error of the rope-driven force sensing flexible gripper is within an acceptable range, which verifies the feasibility and effectiveness of the force sensing device.

## 5. Conclusions

This paper proposed a novel rope-driven force sensing flexible gripper, which can be used for the robotic grasping system and realize the grasping force sensing function without the need for a force sensor. The following conclusions are obtained:

(1) A rope-driven force sensing flexible gripper is designed and the prototype is developed. The force sensing unit and the gripper unit are connected by ropes. When the gripper unit grasps an object, the force sensing function of the grasping object can be realized based on the rope rotation.

(2) A force sensing algorithm and a control system of the rope-driven force sensing flexible gripper is built. The force sensing algorithm is embedded in the lower computer, while the upper computer is used to control and display the results, and the force sensing strategy is built based on the control system.

(3) The experimental system of the rope-driven force sensing flexible gripper is built, and the grasping force sensing strategy is verified through the actual experimental test. The results show that the average error of force sensing through the force sensing strategy is about 2.3%. The rope-driven force sensing flexible gripper proposed can realize the grasping force sensing function for different diameters objects at different contact nodes, which provides a new idea for the design and force sensing method of intelligent robotic grasping systems in robotic teaching, scientific research, and industrial applications.

## Data Availability

The data used in this article can be made available upon reasonable request. Please contact the first author, Y.L. (liuyufeiahpu@126.com).
